# Electromyography and sonomyography analysis of the tibialis anterior: a cross sectional study

**DOI:** 10.1186/1757-1146-7-11

**Published:** 2014-02-08

**Authors:** Maria Ruiz-Muñoz, Antonio I Cuesta-Vargas

**Affiliations:** 1 Nursing and Podiatry Department, Faculty of Health Sciences, University of Malaga, Av/Arquitecto Peñalosa s/n (Teatinos Campus Expansion), 29009 Malaga, Spain; 2 Physiotherapy Department, Faculty of Health Sciences, Biomedical Research Institute of Malaga (IBIMA), University of Malaga, Av/Arquitecto Peñalosa s/n (Teatinos Campus Expansion), 29009 Malaga, Spain; 3 School of Clinical Sciences at Queensland University, Brisbane, Australia

## Abstract

**Background:**

Foot dorsiflexion plays an essential role in both controlling balance and human gait. Electromyography (EMG) and sonomyography (SMG) can provide information on several aspects of muscle function. The aim was to establish the relationship between the EMG and SMG variables during isotonic contractions of foot dorsiflexors.

**Methods:**

Twenty-seven healthy young adults performed the foot dorsiflexion test on a device designed ad hoc. EMG variables were maximum peak and area under the curve. Muscular architecture variables were muscle thickness and pennation angle. Descriptive statistical analysis, inferential analysis and a multivariate linear regression model were carried out. The confidence level was established with a statistically significant *p*-value of less than 0.05.

**Results:**

The correlation between EMG variables and SMG variables was r = 0.462 (*p* < 0.05). The linear regression model to the dependent variable “peak normalized tibialis anterior (TA)” from the independent variables “pennation angle and thickness”, was significant (*p* = 0.002) with an explained variance of R^2^ = 0.693 and SEE = 0.16.

**Conclusions:**

There is a significant relationship and degree of contribution between EMG and SMG variables during isotonic contractions of the TA muscle. Our results suggest that EMG and SMG can be feasible tools for monitoring and assessment of foot dorsiflexors. TA muscle parameterization and assessment is relevant in order to know that increased strength accelerates the recovery of lower limb injuries.

## Background

Foot dorsiflexion plays an essential role in both controlling balance and human gait [1-3]. The correct performance of this movement in concentric and eccentric isotonic contractions is essential for walking and other activities of daily living (ADLs) such as going up and down stairs, walking up slopes and running [4]. The main ankle dorsiflexor muscle is the tibialis anterior (TA), where dysfunction in the activation of the ankle dorsiflexors can result in “dropped foot” [5]. This muscle is also of particular relevance since increases in its strength reduce the risk of falls [6] and accelerate the recovery of lower limb injuries [7].

Most TA muscle activity in walking takes place during the swing phase and during heel strike [7]. During normal gait, the muscles work either as an accelerator, through concentric contraction, or as a shock absorber for deceleration, through eccentric contraction [8]. Any dysfunction of the TA muscle will hinder the lifting of the foot through concentric contraction while the leg swings forward, and will also alter the falling of the foot onto the surface through eccentric contraction after heel strike. This will lead to slower, more inefficient steps whilst walking [8]. Different quantitative methods have contributed to research into the mechanisms of normal and pathological gait and its evaluation [8].

TA muscle activity during foot dorsiflexion is studied using EMG, which is generated by a record of electrical discharges of active motor units during muscle activation [9]. EMG signals can provide information on several aspects of muscle function, such as muscle fatigue [10,11], muscle pathology [10-13] and prosthesis control [14-18].

The muscular architecture of the TA muscle during foot dorsiflexion is studied using sonomyography (SMG), capturing an ultrasound (US) image of changes in muscle morphology [16]. This is used in the evaluation of both isometric [16-18] and isotonic contractions [19,20]. Specifically, SMG can be used to measure changes in muscle thickness [21-23], muscle-fiber pennation angle [22-24], muscle fascicle length [25], muscle size [26] and the muscle cross-sectional area [26,27], among others.

Comparative study of muscular architecture with EMG is becoming ever more widespread since it could provide a safe, non-invasive way of determining the muscular function of the superficial muscles [28,29]. Many of these studies focus on muscle function within traditional predictive musculoskeletal models, although the level of sophistication of models with EMG and SMG is now much improved [29]. There are studies which have developed new variables for assessing and treating musculoskeletal function in different parts of the body. Some authors have focused their studies on the trunk [28,30-37]. Other authors have focused on the role of ADL in the upper limb [17,28,37]. The lower limb is also widely studied, mainly due to its relevance in human gait [38,39]. Research has been carried out into the TA muscle during isometric contractions at different intensities using EMG and SMG [28,38], although no studies on isotonic contractions in this muscle were found. By parameterizing foot dorsiflexion during isotonic contractions and synchronizing EMG and SMG, muscular activity and architecture variables can be studied to facilitate the monitoring of key aspects of this foot movement.

The main aim of this study was to describe a new method for real-time monitoring of muscular activity, as measured using EMG, and muscular architecture, as measured using SMG, during isotonic contractions of the foot dorsiflexors. The second aim was to establish the relationship and level of contribution of SMG variables to muscle activity. We hypothesize that there exists a moderate-to-strong relationship and degree of contribution between EMG and SMG variables [28,38].

## Methods

### Participants

Twenty seven healthy young adults (15 men and 12 women) aged 27.8 ± 5.9 years, 1.72 ± 0.11 meters tall and 69.69 ± 13.12 kg in weight were recruited for this study. Participants aged over 40 years with any type of illness, injury or intervention in the lower limb were excluded from the study, since it is known that anatomical and physiological changes in the skeletal muscle and dysfunction in the locomotor system increase with age [40]. Each of the participants gave informed consent in writing prior to the study. Ethical approval for the study was granted by the Ethics Committee of the Faculty of Health Sciences at the University of Malaga.

### Experimental procedure

**Figure 1:**
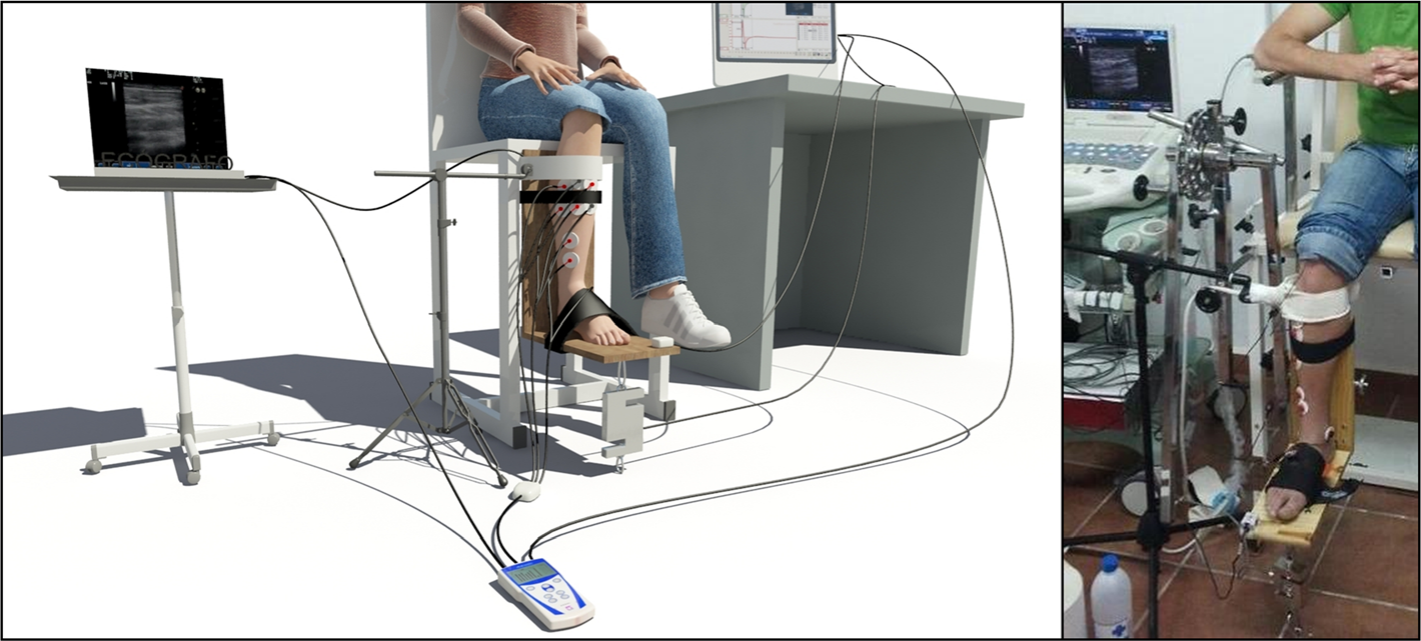
Experimental setup.

A cross-sectional study was designed. The participants sat in a chair which had been specially adapted in line with their size. The location of the chair was established beforehand. The hip and knee were positioned at 90°. A specially designed height-adjustable device comprising two platforms, one vertical and one horizontal, was attached to the chair. The platforms formed an angle of 90°, therefore allowing maximum foot dorsiflexion whilst preventing foot plantar flexion (Figure 1). The sole of the right foot was placed on the horizontal platform, whilst the posterior lower half of the leg was in contact with the vertical platform, forming a maximum angle of 90° between foot and leg. The foot and the lower half of the leg were attached to the device with Velcro straps in order to prevent any changes of position during the test. The bisection of the knee joint and the center of the rotation axis of the load cell had an angle of 0° in the frontal plane and in the sagittal plane measured with a dual-axis goniometer.

The load cell was positioned between the ground and the horizontal platform, and secured to both using ring clamps. A series of non-extendable links allowed the distance to the ground to be adjusted in accordance with the participant’s leg length (Figure 1).

The acquisition and processing signal was carried out under International Society of Electrophysiology and Kinesiology (ISEK) standards [41] and as recommended by Surface Electromyography for the Non-Invasive Assessment of Muscles (SENIAM) [42]. The load cell was taken off, as detailed below.

The skin was shaved and cleaned with alcohol, marking the exact location of the electrodes with a pen. The electrodes were placed, with an interelectrode distance of 20 mm, in the proximal one third of the line running between the proximal end of the fibula and the tip of the medial malleolus, following palpation of the muscle belly of the TA muscle during dorsiflexion and inversion of the foot. Three 5 cm round pregelled Al/AgCl electrodes (Lessa, Barcelona, Spain) [43] were used for each participant and complete procedure (Figure 1).

A free area was left in the TA muscle belly in order to position the ultrasound probe without affecting the position and connection of the electrodes. The probe stayed fixed in the chosen position thanks to an articulated mechanical arm system, which the probe head was placed in, thus allowing its height and angle to be adjusted whilst also preventing it from moving.

After several contractions for the purpose of familiarization, each participant was asked to use the right foot and the tests described below were carried out in the same order:

*Maximal voluntary contraction (MVC):* The maximal isometric foot dorsiflexion used for normalization of the study variables were recorded for each participant. Three maximal isometric dorsiflexion contractions of the right foot were carried out for 5 seconds, with a 90-second rest between each one. An artificial horn was sounded to mark the start of each contraction. Ultrasound signals, electromyographic signals and the force generated by the resistance offered by the load cell secured to the ground and to the horizontal platform were collected during this test.

*Isotonic dorsiflexion contractions:* Isotonic foot dorsiflexion contractions, including concentric and eccentric phases, were carried out without any resistance on the right foot of all participants. This consisted of a dynamic test in which the foot started from a position of 90° relative to the leg, with the participant having to reach the maximal foot dorsiflexion range as quickly as possible (maximum speed). This test also included three consecutive contractions with a 90-second rest between each one. An artificial horn was sounded to mark the start of each contraction. All participants received the same initial instructions with regards to the movement and the same verbal stimuli were given as feedback during each contraction. Electromyographic and ultrasound records were taken during the test. The load cell was taken off to carry out this test.

Before the test protocol, each participant performed as many repetitions of the movement as deemed necessary in order to become familiar with it.

### Data acquisition

The sEMG and SMG data were recorded continuously and synchronously during each test using the Biomonitor ME6000 [44] console with Megawin 3.0.1 software, which all the devices were connected to. Image acquisition was carried out using a duly adapted image capture device and a software add-on (Video EMG Option). The video signal was digitalized with a rate of 24 frames per second (a frame corresponds to the image obtained from the video). This allowed offline searching for the ultrasound image and the electromyographic data for the selected instant. For this study, the maximum muscular activation peak was located and an image was taken in order to subsequently measure the muscular architecture variables (thickness and pennation angle) of the TA muscle.

The start and end of the synchronizing of all systems during each test were marked by an activation device or trigger (DV Trigger Mega Electronics Ltd). The recording of the sEMG and SMG started before the first contraction with the foot in the starting position and stopped when the participants had finished the last contraction and returned to the starting position.

*Electromyographic acquisition:* The electrical activation of the TA muscle was measured using the Biomonitor ME6000 electromyograph (Mega Electronics Ltd, Kuopio, Finland) with a sampling frequency of 1000 Hz.

Raw data were recorded and processed by MegaWin 3.0.1. (Mega Electronics Ltd, Kuopio, Finland) and filtered using a bidirectional fourth-order, 20 Hz low pass Butterworth filter to remove high-frequency noise from the sample. Electrode size, interelectrode distance and location were chosen carefully in order to avoid EMG crosstalk [41].

*Ultrasound acquisition:* Ultrasound images were obtained using the Esaote MyLab25 Gold scanner with a model LA523 probe set to a frequency of 12 MHz [45]. The same operator carried out all acquisitions during the two tests (isotonic dorsiflexion and MVC). TA muscle thickness and pennation angle were acquired by placing the probe on the sagittal plane; in both cases the probe remained just below the tibial tuberosity, parallel to the palpable edge of the tibia [28].

### Data analysis

*EMG data analysis:* The electromyographic variables, maximum peak and area under curve (AUC) were extracted from the basic results for the selected area of interest for all participants. This area includes the maximum activation peak of the electromyographic register of the TA muscle and the two seconds around it (one second before and one second after). The largest maximum activation peak among the three trials performed by each participant was selected for analysis. MegaWin 3.0.1 software was used to obtain these variables (Mega Electronics Ltd, Kuopio, Finland).

**Figure 2:**
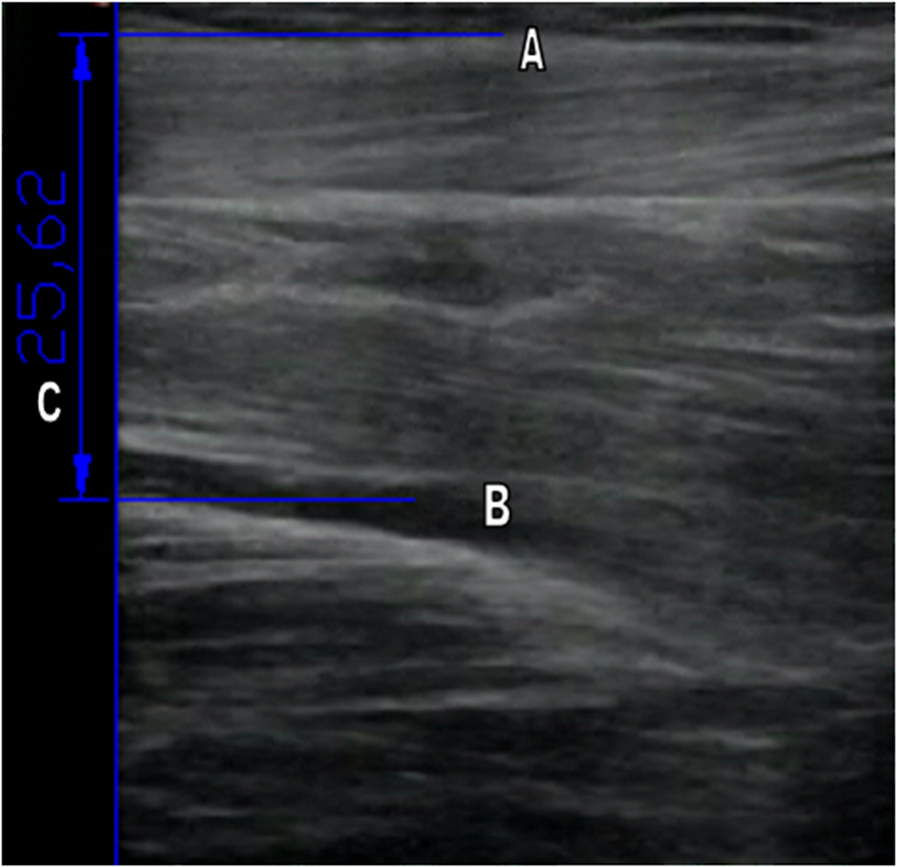
**Measurement of the muscle thickness of TA muscle. A**. Surface edge. **B**. Surface of the tibia. **C**. Muscle thickness.

**Figure 3:**
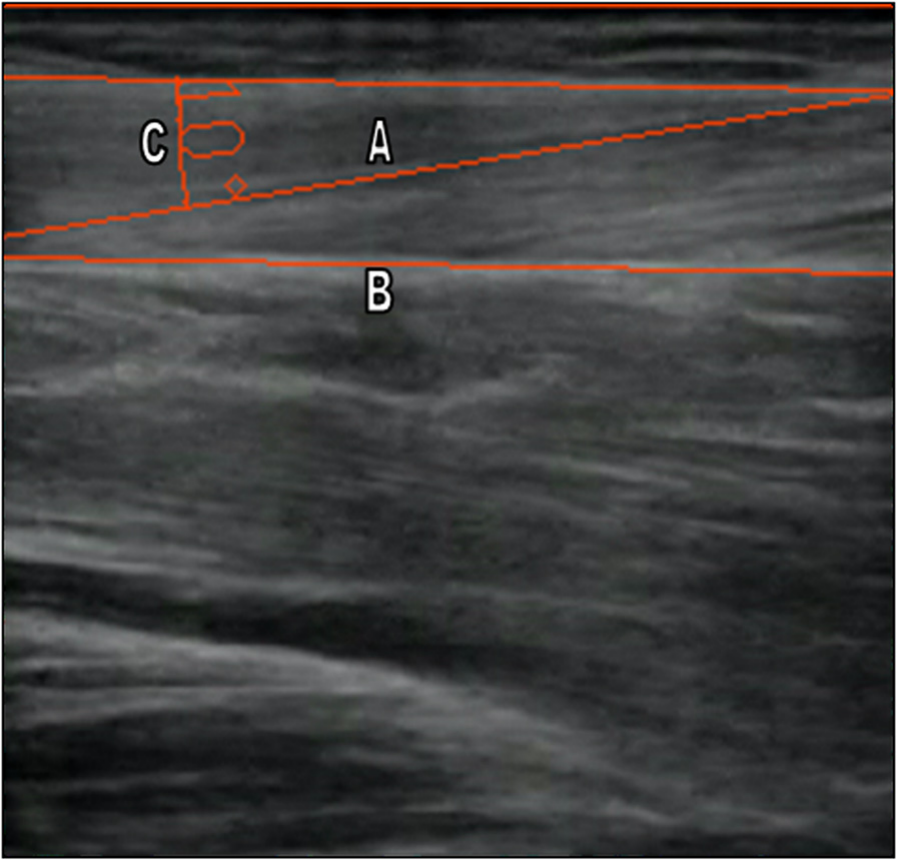
**Image showing the measurement of the pennation angle of TA muscle. A**. Connective tissue. **B**. Surface fascicle **C**. Pennation angle.

*SMG data analysis:* The muscular architecture variables thickness and pennation angle from the ultrasound images were taken following the procedure described by Hodges et al. [28]. Two images for each test and for each participant were analyzed, always in the same order; the first image was muscle thickness and the second was pennation angle. Muscle thickness was measured between the surface edge and the surface of the tibia at 5 cm from the right edge of the image (Figure 2) and the pennation angle was determined from the angle between the connective tissue which runs longitudinally down the center of the muscle and the most clearly shown surface fascicle (Figure 3). Marking for thickness was done through detection of the pixel closest to white using Matlab Program, and for pennation angle the clearest muscle fiber was selected. Muscular architecture variables were obtained from the images extracted from the video captured during synchronous measurement, and were measured offline. F.205.0.0 AutoCAD 2012-English SP2 software (Autodesk, San Rafael, California, USA) was used to extract these parameters.

Two measurements of each single image performed by the same operator on the same day, corresponding to the maximum peaks of muscular activity carried out during the test, were used to evaluate the reliability of the measurement. The operator did not know the measurement calculated in the first measurement.

The values extracted from the electromyographic records and the muscular architecture obtained in the isotonic dorsiflexion contractions were normalized for each participant with regards to the maximum values obtained in the MVC.

**Figure 4:**
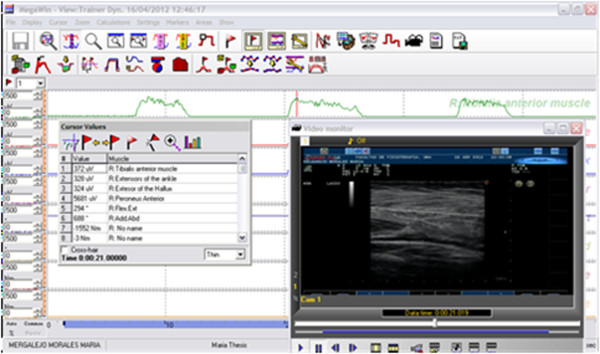
Synchronization example of ultrasound images and sEMG muscle activity.

Examples of muscular architectural parameters for ultrasound synchronized with sEMG muscle activity for one participant are shown in Figure 4.

### Statistical analysis

Statistical analysis was carried out with SPSS 15.0 for Windows. The confidence level was established with a statistically significant *p*-value of less than 0.05. All variables were inspected and confirmed for a normal distribution prior to analysis (Kolmogorov**–**Smirnow normality test). Reliability analysis was studied using the Intraclass Correlation Coefficient (ICC) with a confidence interval of 95%.

The Pearson’s correlation coefficient (*r*) was calculated between EMG and SMG variables. Linear regressions were performed between the dependent variable “peak normalized TA” from the independent variables “pennation angle and thickness”. The magnitude of the correlations was considered as being <0.3 weak, 0.3 to 0.5 moderate and 0.5 to 0.8 strong [46,47].

## Results

Descriptive data from EMG and ultrasound images (27 participants) is shown in Table 1. Reliability results for measurements of muscle architecture variables were muscle thickness ICC = 0.991 (0.979 to 0.996) and pennation angle ICC = 0.910 (0.795 to 0.960).

**Table 1:** Descriptive data from EMG and SMG variables of TA muscle

	**Isotonic %MVC normalized mean (SD)**	**Isotonic %MVC units mean (SD)**
Maximum peak EMG TA	0.737 (0.208)	411.8 (162.6) (uV)
Area EMG TA	0.419 (0.394)	290.54 (246.7) (uV)
Thickness TA	0.927 (0.291)	19.75 (2.63) (mm)
Pennation angle TA	0.828 (0.432)	9.60 (1.4) (degrees)

MVC: maximal voluntary contraction.

EMG: electromyography.

TA: tibialis anterior.

Results of the correlations between the EMG and SMG variables are shown in Table 2. The linear regression model to the dependent variable “peak normalized TA” from the independent variables “pennation angle and thickness” was significant (F = 8.795; *p* = 0.002) with an explained variance of R^2^ = 0.693 and SEE = 0.16. The contribution of each predictor to the model was significant with a standardized Beta coefficient of 0.510 (*p* = 0.006) and 0.519 (*p* = 0.006) respectively. Moreover, the model explained 69% of the variance.

**Table 2:** Correlations between EMG and SMG data normalized with regards to the maximum values obtained in the MVC from TA

	**Thickness**	**Pennation angle**	**Maximum peak EMG**
Pennation angle	r = -0.091		
	(p = 0.687)		
Maximum peak EMG	r = 0.473	r = 0.462	
	(p = 0.023)	(p = 0.030)	
Area EMG	r = 0.025	r = 0.364	r = 0.267
	(p = 0.911)	(p = 0.096)	(p = 0.197)

MVC: maximal voluntary contraction.

TA: tibialis anterior.

EMG: electromyography.

*r* = Pearson’s *r* correlation coefficient.

*p* = statistical significance.

## Discussion

Our findings suggest that the present method may be clinically relevant because it involves a reproducible procedure, which allows the function and structure of the foot dorsiflexors to be monitored. TA muscle parameterization and assessment is relevant in order to know that increased strength reduces the risk of falls [6] and accelerates the recovery of lower limb injuries [7]. This synchronized recording method may be extended not only to diagnosis but also to the evaluation of therapies, as has been done for other muscles, movements and gestures [19,21,30,48,49].

This study showed that there is a moderate-to-strong correlation between the EMG activity of the muscle and the architectural variables muscle thickness and pennation angle for the TA muscle. In other words, thickness and pennation angle increase proportionally as electric activity increases during isotonic contractions of the TA muscle.

Pennation angle of the muscle and muscle thickness explain 69% of the variance of the EMG maximum peak reached by the TA muscle during isotonic contractions. Both predictor variables make up around 50% of the model, demonstrating a high degree of relation. The remaining unexplained variance for this model may be related to the intrinsic drawbacks of using sEMG measurement, including the properties of the underlying tissues such as muscle fiber or fat type, subcutaneous tissue thickness or skin characteristics [50]. These drawbacks include interference or cross-talk from adjacent muscles [28] and the positioning of electrodes.

Furthermore, this study includes analysis of the reliability of the SMG measurement of both the thickness and the pennation angle of the TA muscle, resulting in ICC = 0.991 (0.979 to 0.996) and ICC = 0.910 (0.795 to 0.960) respectively, which is consistent with other similar measurement systems [22].

To our knowledge, this is the first study to link sEMG and SMG in the TA muscle during foot dorsiflexion in an isotonic test at maximum speed. Other studies have found similar relations between the same variables during isometric contractions in the TA muscle and in other regions. Two studies found a strong relation (R^2^ = 0.76) [38] and (R^2^ = 0.96) [28] between EMG and the pennation angle of the TA muscle during isometric contractions. A strong relation (R^2^ = 0.75) was also found between EMG and muscle thickness [28]. Moreover, another study analyzed the changes which came about in the pennation angle from rest in the TA muscle during maximal isometric contraction, finding changes above 60% [51].

Similar studies, all involving isometric tests in other regions of the lower limb, found a significant relation between EMG and pennation angle in the leg muscles, soleus, lateral and medial gastrocnemius (r > 0.80) [38]. Thigh muscles such as the rectus femoris muscle also showed a strong correlation (R^2^ = 0.999), but in this case between SMG and torque [39].

However, the study carried out by Maganaris and Baltzopoulos [51] did not show any significant changes in TA thickness during MVC, which may be because, unlike other studies [28], the test was carried out from 15° of ankle dorsiflexion (rest or initial condition) to 30° of plantar flexion (MVC or final condition). Similarly, Ghori et al. [50] did not find any relation between vastus lateralis muscle EMG and torque, although there were notable differences in the procedure (the test consisted of five consecutive concentric and eccentric contractions) compared to those studies which found a relationship between EMG and torque in other quadriceps muscles [39] (this case involved two tests: step contraction, with sub-maximal MVC torque, and ramp increasing and decreasing).

There are numerous studies which link muscle architecture and electrical activity in muscles in upper limbs. Shi et al. [17,52,53] carried out several studies which focused on the arm and forearm muscles and found moderate-to-strong correlations between sonographic and electromyographic variables; other authors in similar studies achieved the same results [20,37].

There are several limitations regarding the present study. Participants were healthy subjects, preventing comparison between healthy and diseased. There is also the possibility of EMG crosstalk, even though electrode size, distance and location were chosen carefully to prevent this. Moreover, some of the unexplained variance in the model could be due to speed variability during the test of each participant.

## Conclusions

Our results suggest that real-time monitoring of EMG and SMG can be a feasible tool for the parameterization and optimal assessment of foot dorsiflexion. There is a significant relationship and degree of contribution between EMG and SMG variables during isotonic contractions of the TA muscle.

Moreover, future studies should include groups of participants with impaired foot function and structure, in order to establish sub-groups based on the direct variables obtained during the procedure described in this study, and also to develop new indices which combine the most discriminate variables or those which are sensitive to change.

## Competing interests

The authors declare that they have no competing interests.

## Authors’ contributions

MRM carried out the acquisition, analysis and interpretation of data, performed the statistical analysis and drafted the manuscript. AICV conceived the study, participated in its design performed the statistical analysis and drafted the manuscript. All authors read and approved the final manuscript.

## References

[B1] Mann RA Hagy J Biomechanics of walking, running, and sprinting Am J Sports Med 1980 8 345 350 10.1177/036354658000800510 7416353

[B2] Cornwall MW McPoil TG The influence of tibialis anterior muscle activity on rearfoot motion during walking Foot Ankle Int 1994 15 75 79 10.1177/107110079401500205 7981805

[B3] Daubney ME Culham EG Lower-extremity muscle force and balance performance in adults aged 65 years and older Phys Ther 1999 79 1177 1185 10630286

[B4] Nilsson J Thorstensson A Halbertsma J Changes in leg movements and muscle activity with speed of locomotion and mode of progression in humans Acta Physiol Scand 1985 123 457 475 10.1111/j.1748-1716.1985.tb07612.x 3993402

[B5] Burridge J Taylor P Hagan S Swain I Experience of clinical use of the Odstock dropped foot stimulator Artif Organs 1997 21 254 260 10.1111/j.1525-1594.1997.tb04662.x 9148719

[B6] Lee SE Effects of increasing ankle range of motion program on ambulation and balance for the elderly with balance disorder J Korean Acad Univ Trained Phys Ther 2005 12 28 36

[B7] Woollacott MH Shumway-Cook A Nashner LM Aging and posture control: changes in sensory organization and muscular coordination Int J Aging Hum Dev 1986 23 97 114 10.2190/VXN3-N3RT-54JB-X16X 3557634

[B8] Dobkin BH The Clinical Science of Neurologic Rehabilitation 2003 Oxford: Oxford University Press

[B9] Zwarts MJ Stegeman DF Multichannel surface EMG: basic aspects and clinical utility Muscle Nerve 2003 28 1 17 10.1002/mus.10358 12811768

[B10] Fukuda K Umezu Y Shiba N Tajima F Nagata K Electromyographic fatigue analysis of back muscles during remote muscle contraction J Back Musculoskelet Rehabil 2006 19 61 66

[B11] Haig AJ Gelblum JB Rechtien JJ Gitter AJ Technology assessment: the use of surface EMG in the diagnosis and treatment of nerve and muscle disorders Muscle Nerve 1996 19 392 395 10.1002/(SICI)1097-4598(199603)19:3<392::AID-MUS21>3.0.CO;2-T 8606710

[B12] Masuda K Masuda T Sadoyama T Inaki M Katsuta S Changes in surface EMG parameters during static and dynamic fatiguing contractions J Electromyogr Kinesiol 1999 9 39 46 10.1016/S1050-6411(98)00021-2 10022560

[B13] Hogrel JY Clinical applications of surface electromyography in neuromuscular disorders Neurophysiol Clin 2005 35 59 71 10.1016/j.neucli.2005.03.001 16087069

[B14] Boostani R Moradi MH Evaluation of the forearm EMG signal features for the control of a prosthetic hand Physiol Meas 2003 24 309 319 10.1088/0967-3334/24/2/307 12812417

[B15] Soares A Andrade A Lamounier E Carrijo R The development of a virtual myoelectric prosthesis controlled by an EMG pattern recognition system based on neural networks J Intell Inf Syst 2003 21 127 141 10.1023/A:1024758415877

[B16] Shi J Zheng YP Chen X Huang QH Assessment of muscle fatigue using sonomyography: muscle thickness change detected from ultrasound images Med Eng Phys 2007 29 472 479 10.1016/j.medengphy.2006.07.004 16908212

[B17] Shi J Zheng Y-P Huang Q-H Chen X Continuous monitoring of sonomyography, electromyography and torque generated by normal upper arm muscles during isometric contraction: sonomyography assessment for arm muscles IEEE Trans Biomed Eng 2008 55 1191 1198 18334413 10.1109/TBME.2007.909538

[B18] Zhou Y Zheng Y-P Estimation of muscle fiber orientation in ultrasound images using revoting hough transform (RVHT) Ultrasound Med Bio 2008 34 1474 1481 10.1016/j.ultrasmedbio.2008.02.009 18420336

[B19] Guo J-Y Zheng Y-P Huang Q-H Chen X Dynamic monitoring of forearm muscles using one-dimensional sonomyography system J Rehabil Res Dev 2008 45 187 195 10.1682/JRRD.2007.02.0026 18566937

[B20] Xie H-B Zheng Y-P Guo J-Y Chen X Shi J Estimation of wrist angle from sonomyography using support vector machine and artificial neural network models Med Eng Phys 2009 31 384 391 10.1016/j.medengphy.2008.05.005 18586548

[B21] Zheng YP Chan MMF Shi J Chen X Huang QH Sonomyography: monitoring morphological changes of forearm muscles in actions with the feasibility for the control of powered prosthesis Med Eng Phys 2006 28 405 415 10.1016/j.medengphy.2005.07.012 16115790

[B22] Brancaccio P Limongelli FM D’Aponte A Narici M Maffulli N Changes in skeletal muscle architecture following a cycloergometer test to exhaustion in athletes J Sci Med Sport 2008 11 538 541 10.1016/j.jsams.2007.05.011 17905658

[B23] Legerlotz K Smith HK Hing WA Variation and reliability of ultrasonographic quantification of the architecture of the medial gastrocnemius muscle in young children Clin Physiol Funct Imaging 2010 30 198 205 10.1111/j.1475-097X.2010.00925.x 20184623

[B24] Rutherford OM Jones DA Measurement of fiber pennation using ultrasound in the human quadriceps in vivo Eur J Appl Physiol Occup Physiol 1992 65 433 437 10.1007/BF00243510 1425649

[B25] Fukunaga T Ichinose Y Ito M Kawakami Y Fukashiro S Determination of fascicle length and pennation in a contracting human muscle in vivo J Appl Physiol (1985) 1997 82 354 358 9029238 10.1152/jappl.1997.82.1.354

[B26] Reeves ND Maganaris CN Narici MV Ultrasonographic assessment of human skeletal muscle size Eur J Appl Physiol 2004 91 116 118 10.1007/s00421-003-0961-9 14639480

[B27] Fukunaga T Kawakami Y Kuno S Funato K Fukashiro S Muscle architecture and function in humans J Biomech 1997 30 457 463 10.1016/S0021-9290(96)00171-6 9109557

[B28] Hodges PW Pengel LHM Herbert RD Gandevia SC Measurement of muscle contraction with ultrasound imaging Muscle Nerve 2003 27 682 692 10.1002/mus.10375 12766979

[B29] Lloyd D EMG-Assisted neuromuscular skeletal modelling: going beyond emg to assess the action of muscles in movement Proceedings of the XIXth Congress of the International Society of Electrophysiology & Kinesiology: 19-21 July 2012 Brisbane, Australia 31

[B30] Whittaker JL Warner MB Stokes M Comparison of the sonographic features of the abdominal wall muscles and connective tissues in individuals with and without lumbopelvic pain J Orthop Sports Phys Ther 2013 43 11 19 10.2519/jospt.2013.4450 23160368

[B31] Brown SHM McGill SM A comparison of ultrasound and electromyography measures of force and activation to examine the mechanics of abdominal wall contraction Clin Biomech (Bristol, Avon) 2010 25 115 123 10.1016/j.clinbiomech.2009.10.001 19879679

[B32] Stokes M Hides J Elliott J Kiesel K Hodges P Rehabilitative ultrasound imaging of the posterior paraspinal muscles J Orthop Sports Phys Ther 2007 37 581 595 10.2519/jospt.2007.2599 17970405

[B33] Masuda T Miyamoto K Oguri K Matsuoka T Shimizu K Relationship between the thickness and hemodynamics of the erector spinae muscles in various lumbar curvatures Clin Biomech (Bristol, Avon) 2005 20 247 253 10.1016/j.clinbiomech.2004.10.008 15698696

[B34] Watanabe K Miyamoto K Masuda T Shimizu K Use of ultrasonography to evaluate thickness of the erector spinae muscle in maximum flexion and extension of the lumbar spine Spine 2004 29 1472 1477 10.1097/01.BRS.0000128755.84693.10 15223941

[B35] Masuda T Miyamoto K Shimizu K Intramuscular hemodynamics in bilateral erector spinae muscles in symmetrical and asymmetrical postures with and without loading Clin Biomech (Bristol, Avon) 2006 21 245 253 10.1016/j.clinbiomech.2005.10.008 16364517

[B36] Cuesta-Vargas AI Gonález-Sánchez M Relationship of moderate and low isometric lumbar extension through architectural and muscular activity variables: a cross sectional study BMC Med Imaging 2013 13 38 10.1186/1471-2342-13-38 24252273 PMC3840670

[B37] Huang QH Zheng YP Chena X He JF Shi J A system for the synchronized recording of sonomyography, electromyography and joint angle Open Biomed Eng J 2007 1 77 84 10.2174/1874120700701010077 19662132 PMC2701082

[B38] Manal K Roberts DP Buchanan TS Can pennation angles be predicted from EMGs for the primary ankle plantar and dorsiflexors during isometric contractions? J Biomech 2008 41 2492 2497 10.1016/j.jbiomech.2008.05.005 18579147 PMC2548308

[B39] Guo J-Y Zheng Y-P Xie H-B Chen X Continuous monitoring of electromyography (EMG), mechanomyography (MMG), sonomyography (SMG) and torque output during ramp and step isometric contractions Med Eng Phys 2010 32 1032 1042 10.1016/j.medengphy.2010.07.004 20688554

[B40] Kim EJ Kim TS Bae SS Fall-related injury and balance of the elderly J Korean Acad Univ Trained Phys Ther 1998 10 161 171

[B41] ISEK Standards for reporting EMG data http://www.isek-online.org/standards_emg.html

[B42] SENIAM http://www.seniam.org/

[B43] Lessa - productos http://www.lessap.com/productos.htm

[B44] ME6000 | Mega electronics Ltd http://www.megaemg.com/products/biomonitor-me6000/

[B45] Esaote espña http://www.esaote.es/modules/core/page.asp?p=MYLAB25_GOLD&t=OVE

[B46] Cohen J Statistical Power Analysis for the Behavioral Sciences 1988 New Jersey: Lawrence Erlbaum Associates

[B47] Cohen J Statistical Power Analysis for the Behavioral Sciences 1987 Hillsdale, New Jersey: L. Erlbaum Associates

[B48] Guo J-Y Zheng Y-P Kenney LP Xie H-B Evaluation of sonomyography (SMG) for control compared with electromyography (EMG) in a discrete target tracking task Conf Proc IEEE Eng Med Biol Soc 2009 2009 1549 1552 19963507 10.1109/IEMBS.2009.5332398

[B49] Shi J Chang Q Zheng Y-P Feasibility of controlling prosthetic hand using sonomyography signal in real time: preliminary study J Rehabil Res Dev 2010 47 87 98 10.1682/JRRD.2009.03.0031 20593322

[B50] Ghori GM Donne B Luckwill RG Relationship between torque and EMG activity of a knee extensor muscle during isokinetic concentric and eccentric actions J Electromyogr Kinesiol 1995 5 109 115 10.1016/1050-6411(94)00013-C 20719642

[B51] Maganaris CN Baltzopoulos V Predictability of in vivo changes in pennation angle of human tibialis anterior muscle from rest to maximum isometric dorsiflexion Eur J Appl Physiol Occup Physiol 1999 79 294 297 10.1007/s004210050510 10048637

[B52] Shi J Zheng Y Yan Z Huang Q Preliminary study of skeletal muscle with multi-signals during isometric contraction Conf Proc IEEE Eng Med Biol Soc 2006 1 5080 5083 17946285 10.1109/IEMBS.2006.259705

[B53] Shi J Zheng Y Yan Z SVM for estimation of wrist angle from sonomyography and SEMG signals Conf Proc IEEE Eng Med Biol Soc 2007 2007 4806 4809 18003081 10.1109/IEMBS.2007.4353415

